# A Comparison of Growth on Mercuric Chloride for Three *Lemnaceae* Species Reveals Differences in Growth Dynamics That Effect Their Suitability for Use in Either Monitoring or Remediating Ecosystems Contaminated With Mercury

**DOI:** 10.3389/fchem.2018.00112

**Published:** 2018-04-16

**Authors:** Jingjing Yang, Gaojie Li, Anthony Bishopp, P. P. M. Heenatigala, Shiqi Hu, Yan Chen, Zhigang Wu, Sunjeet Kumar, Pengfei Duan, Lunguang Yao, Hongwei Hou

**Affiliations:** ^1^The State Key Laboratory of Freshwater Ecology and Biotechnology, The Key Laboratory of Aquatic Biodiversity and Conservation of Chinese Academy of Sciences, Institute of Hydrobiology, Chinese Academy of Sciences, University of Chinese Academy of Sciences, Wuhan, China; ^2^Centre for Plant Integrative Biology, University of Nottingham, Nottingham, United Kingdom; ^3^Collaborative Innovation Center of Water Security for Water Source Region of Mid-line of South-to-North Diversion Project, College of Agricultural Engineering, Nanyang Normal University, Henan, China

**Keywords:** duckweed, mercuric chloride, toxicity test, growth indices, chemical composition, biomonitoring, bioremediation

## Abstract

Mercury (Hg) is a toxic heavy metal that can alter the ecological balance when it contaminates aquatic ecosystems. Previously, researchers have used various *Lemnaceae* species either to monitor and/or remove heavy metals from freshwater systems. As Hg contamination is a pressing issue for aquatic systems worldwide, we assessed its impact on the growth of three commonly species of *Lemnaceae*- *Lemna gibba* 6745*, Lemna minor* 6580 and *Spirodela polyrhiza* 5543. We exposed plants to different concentrations of mercuric chloride (HgCl_2_) and monitored their growth, including relative growth rate, frond number (FN), and fresh weight (FW). These data were coupled with measurements of starch content, levels of photosynthetic pigment and the activities of antioxidant substances. The growth of all three lines showed significant negative correlations with Hg concentrations, and starch content, photosynthetic pigment, soluble protein and antioxidant enzymes levels were all clearly affected. Our results indicate that the *L. gibba* line used in this study was the most suitable of the three for biomonitoring of water contaminated with Hg. Accumulation of Hg was highest in the *S. polyrhiza* line with a bioconcentration factor over 1,000, making this line the most suitable of the three tested for use in an Hg bioremediation system.

## Introduction

Mercury (Hg) is a toxic heavy metal element (Nieboer and Richardson, 1980; Fitzgerald and Clarkson, 1991). It can have devastating effects on organisms as well as on the whole environment when it contaminated aquatic ecosystems (Nagajyoti et al., 2010). Both natural and anthropogenic sources cause the accumulation of Hg in aquatic ecosystems. Natural sources include geologic parent material, rock outcroppings, wind-blown dusts, volcanic eruptions, marine aerosols and forest fires whilst anthropogenic sources include mining, coal burning and unsafe disposal of industrial solid/liquid wastes. Modern industrialization and urbanization led to the releases of Hg into ecosystems throughout the world (Sznopek and Goonan, 2000; Kolker et al., 2006; Larssen, 2010). A survey published in 2016 revealed that manufacturing activities in China released, 633 t of Hg emissions to the air, 84 t to water and 651 t to the land (Hui et al., 2017). This increasing contamination of Hg has led to a substantial accumulation within organisms. For example, a recent study reported high levels of methylmercury (MeHg) in the Bohai Sea, China, with some samples exceeding the Grade I limit established in China's seawater quality standard (50 ng/L) (Tong et al., 2017). Based on assessment of Hg contamination in China's coastal waters, MeHg concentrations in human blood were predicted to be between 1.37 and 2.77 mg/L for pregnant women and 0.43–1.00 mg/L for infants (Tong et al., 2017). Such levels affect human health and necessitate restrictions on seafood in the diet. This is a global problem and high Hg accumulation has even been observed in bats in the United States (Korstian et al., 2017). Discharge of industrial waste can also cause rapid increases in Hg. For example, discharge into a reservoir at the lower Ebro River in Catalonia (Spain) resulted in accumulation of Hg 20 times higher than the typical local concentration. Consequently, the total Hg (THg) and methylmercury (MeHg) content in zebra mussels collected in near sites were significantly elevated (Carrasco et al., 2008).

As Hg has such serious effects, considerable work has been done to minimize its discharge into both drinking and wastewater systems, and to maintain Hg levels below an established threshold (Ritter and Bibler, 1990). However, as Hg accumulates within the environment it is necessary to remove it from contaminated water bodies even though this is an expensive process (Pérez-Sanz et al., 2012). Whilst conventional methods such as ion exchange, membrane filtration, chelate precipitation, precipitation/adsorption are effective for aquatic ecosystems, the high costs often prevent their widespread deployment (Jeon and Park, 2005; Unlü and Ersoz, 2006; Wu et al., 2007). Therefore, there is a great need for alternative more cost—efficient methods to evaluate the contamination of Hg in aquatic ecosystems and to remediate these systems.

Plant based bioassays offer an attractive low cost solution to determine the effects and hazards of certain pollutants or environmental factors (Singh et al., 2007) and can provide convenient guidance for biomonitoring and bioremediation (Lewis and Wang, 1997; Roussel et al., 2000). Such processes rely on plants that have ability to accumulate certain substance (Tangahu et al., 2011). Recently there has been great interest in the use of duckweeds for both biomonitoring and bioremediation. Duckweeds are a small group of free-floating aquatic plants belonging to the Lemnaceae family. Members are commonly found in freshwater habitats such as ponds, lakes, ditches and rice paddies (Landolt, 1986). Due to a suite of properties including, their rapid growth rate, their ease of cultivation, the direct contact that they have with the water, their ability to adapt to environmental changes and their significant potential for both metal and nutrient uptake, duckweeds are becoming an attractive group of plants in various biotechnological applications (Lemon et al., 2001; Appenroth et al., 2013). Although the family contains five genera with 37 species, three species: *Lemna gibba, Lemna minor* and *Spirodela polyrhiza* have been studied extensively (Appenroth et al., 2013; Borisjuk et al., 2015; Forni and Tommasi, 2015). The use of duckweeds in such assays has become so widespread that standardized guidelines have been established to evaluate metal toxicity as well as removal of metal contaminants (Day and Saunders, 2004; Reinhold and Saunders, 2006; Tront et al., 2007). For example, ISO 20079 and OECD protocols provide detailed descriptions on the determination of toxicity effect of certain substances or polluted water on *L. gibba* or *L. minor* (Zayed et al., 1998; ISO 20079, 2005; OECD, 2006).

Numerous studies have already been conducted to assess the toxic effect of heavy metals on different duckweed species (Lakatos et al., 1993; Lahive et al., 2011; Leblebici and Aksoy, 2011; Appenroth et al., 2013; Gür et al., 2016). Such studies have proved instrumental in exploring the possibility of utilizing duckweeds as either biomarkers or in bioremediation. Whilst there is a substantial body of evidence assessing the effects of toxicity on duckweed growth of many elements, studies investigating the effect of mercury on duckweed growth are more limited. Li et al. (2011) have reported that water comprising inorganic and organic mercury at the concentration of 12.0 and 50.0 μg/L showed considerable reduction in the concentration of Hg after 40 min treatment with powdered *L. minor*. This processes resulted in treated water that was below both the maximum permitted concentration of Hg in drinking water (1.0 μg/L) and the permitted discharge limit of wastewater (10.0 μgL/L) set by China and USEPA. A comparative study showed that antioxidative enzymes can be activated within 24 h exposure to Hg although these enzymes are activated at lower Hg concentrations in *L. gibba* than *L. minor* (Varga et al., 2013). However, to date there has been no extensive study providing side-by-side comparisons of different duckweed species under various Hg concentrations.

Collectively these studies provide a detailed understanding about the impact of several potential contaminants on duckweed growth. This information could be exploited to produce new methods for biomonitoring or remediation, for example by those involved in environmental management, risk assessment and policy development. However, to fully exploit such a system for biomonitoring and bioremediation of Hg, several areas need to be addressed, these include (1) Establishing a quantitative description of toxic effect of Hg on duckweeds within a specific time range, (2) Observation of the specific response of duckweeds to Hg stress, (3) Comparison of Hg absorption and uptake by different duckweeds, and (4) Identification of suitable duckweeds for Hg biomonitoring and bioremediation.

Within the biosphere, Hg is cycled between three oxidation states of Hg (0, I, and II; Barbosa et al., 2001). The majority of Hg exists in the form of inorganic mercuric salts (HgCl_2_, Hg(OH)_2_, HgS) and organomercurics (MeHg) (USEPA, 1997). Ionic mercury (Hg^2+^) is the predominant form that can be absorbed and taken up by plants (Han et al., 2006) and therefore frequently accumulates in aquatic organisms (Pan and Wang, 2004). Mercuric chloride (HgCl_2_) was used in this study since dissolves in water with relative ease. The toxicity of chlorine ions from HgCl_2_ was not considered in this study because of its high content in cultivation medium and its negligible toxic effect compared to Hg. Lines of three duckweed species (*L. gibba, L. minor*, and *S. polyrhiza*) were chosen based on their widespread distribution and applicability for a toxicology experiment.

These three lines were grown under different concentrations of Hg, and growth assessed using existing methodology defined by the ISO 20079 guidelines. In addition we assayed other parameters to measure fitness including starch content, photosynthetic pigment, levels of antioxidant substances and Hg accumulation. Based on these data, we propose that different duckweed lines that can fulfill different roles in both biomonitoring and bioremediation of aquatic ecosystems contaminated with Hg.

## Materials and methods

### Duckweeds culture and toxicity tests

*Spirodela polyrhiza* (L.) Schleid (5543) was collected from East Lake (N 30°32′, E 114°21′) at the city of Wuhan, Hubei Province, China. *Lemna minor* L. (6580 Harrington, Bergen Co., NJ, USA) and *Lemna gibba* L. (6745 Jacksonville, Tuolumn Co., CA, USA; Bog et al., 2010) were a gift from Prof. Hai Zhao, Chengdu Institute of Biology, Chinese Academy of Sciences. Current toxicity tests were conducted according to the ISO 20079 criteria (ISO 20079, 2005) using modified *Steinberg* medium (Naumann et al., 2007). The composition of *Steinberg* medium was 3.46 mM KNO_3_, 1.25 mM Ca(NO_3_)_2_·4H_2_O, 0.66 mM KH_2_PO_4_, 0.072 mM K_2_HPO_4_, 0.41 mM MgSO_4_·7H_2_O, 1.94 μM H_3_BO_3_, 0.63 μM ZnSO_4_·7H_2_O, 0.18 μM Na_2_MoO_4_·2H_2_O, 0.91 μM MnCl_2_·4H_2_O, 2.81 FeCl_3_·6H_2_O, 4.03 mM EDTANa2. Pre-cleaned 500 mL Erlenmeyer flasks containing 100 mL sterilized *Steinberg* medium (pH 5.5 ± 0.2) were supplemented with 7 different concentrations of HgCl_2_ (Sigma-Aldrich, purity >99%)-−0, 0.25, 0.5, 1, 2, 4, 8 mg/L or 0, 0.92, 1.84, 3.68, 7.37, 14.73, 29.47 μM. This concentration gradient was based on preliminary data from a 24 h acute toxicity test and set based on a geometric scale. 12 axenic fronds (3 colonies each for *L. gibba* and *L. minor*, 4 colonies for *S. polyrhiza*) per flask were added to the culture solution. Colonies were selected with roughly equal sizes from the pre-cultivated axenic stocks and used to inoculate cultures. All toxicity experiments were conducted at 24 ± 2°C under continuous white light at 85 μmol m^−2^s^−1^ and lasted for 7 days. Experiments were performed in triplicate to allow statistical analyses of results.

### Growth measurements

The frond number (FN) (all visible fronds) in each flask was recorded daily throughout the experiment. The fresh weight (FW) was recorded at the beginning and end of the experiment. The calculations of growth rate followed standardized procedures described in ISO 20079 criteria (ISO 20079, 2005).

### Chemical composition of duckweeds

To determine soluble protein and antioxidative enzymes, fresh plant materials (0.05 g) were homogenized in ice with 0.5 mL phosphate saline buffer (pH 7.4, 0.1 M) using a glass homogenizer. Homogenized samples were centrifuged at 3,500 rpm for 20 min. This supernatant was used to determine the content of soluble protein and activities of antioxidative enzymes (total superoxide dismutase, catalase, peroxidase) using commercially available test kits (Nanjing Jiancheng Bioengineering Institute, Nanjing, China; Li et al., 2013; Yan et al., 2013). The absorbance of the supernatant was detected by BioDrop uLite (80-3006-51) under visible light at different wavelengths.

Photosynthetic pigments of duckweeds were extracted in 80% chilled acetone in the dark and estimated as described by Porra et al. (1989). Starch extraction and quantification were done according to the method described by Magel (1991). Starch was extracted with 18% (w/v) HCl. Detection was conducted using 0.5% (w/v) KI and 0.25% (w/v) I_2_ and measured at 605 nm and 530 nm. To determine the Hg content in duckweeds, plant material was dried at 75°C and digested with 10 ml concentrated HNO_3_ acid with the help of microwave digestion system (Anton paar, Multiwave 3000). Digested samples were diluted up to 10 ml with ultra-deionized water. Final concentrations of K_2_Cr_2_O_7_ and HNO_3_ of the samples were adjusted to be within 0.05% (M/V) and 0.05% (V/V) respectively. The residual level of Hg in each sample was measured using Atomic Fluorescence Spectrometer (Analytikjena, ContrAA 700) at the Center of Analysis and Test Center of Wuhan University.

### Statistical analysis

All measurements were conducted using independent plant samples. The SPSS statistical programme (version 18.0) was used for statistical analysis (including variance tests, determining EC50 values and the corresponding 95% confidence intervals, probit regression analysis, specifying the corresponding fitting coefficient (R^2^), and one-way ANOVA analysis). Tukey tests were performed to determine the significance differences among treatments. Values presented in this manuscript are means ± SDs.

## Results

### Growth of duckweeds

**Figure 1:**
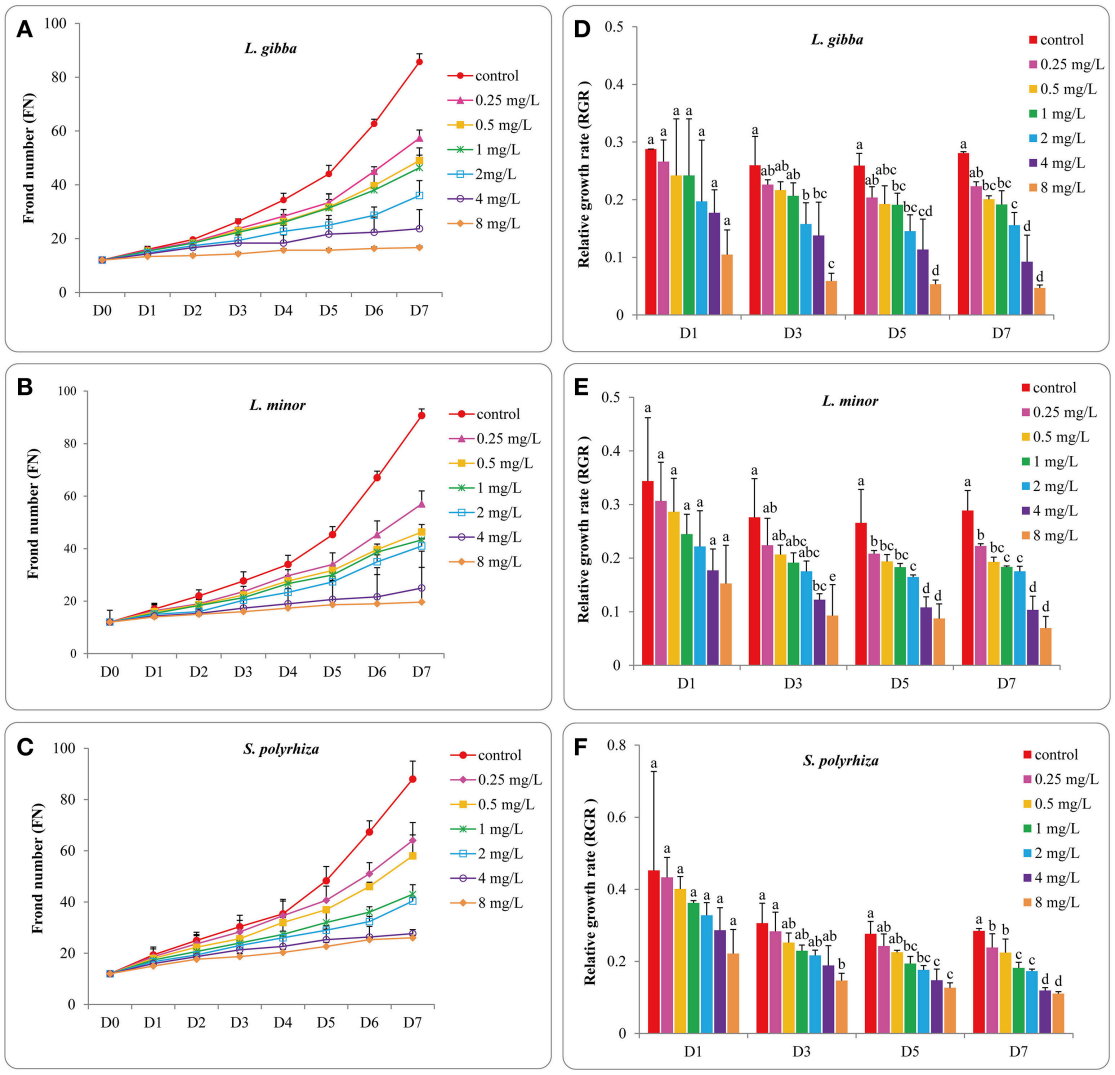
Effects of different concentrations of Hg on the frond number and the corresponding relative growth rates of *L. gibba* **(A,D)**, *L. minor* **(B,E)** and *S. polyrhiza* **(C,F)** at 1, 3, 5, 7 days. The letters (a, b, c, d, e) on the column graphs indicated Tukey tests analyses results among different Hg treatments at 1, 3, 5, 7 days in the same duckweed. The same letters indicated no significant differences and different letters indicated significant difference among treatments. Error bars indicated standard deviation.

To provide a baseline for subsequent toxicity experiments the growth of three duckweed lines was first measured in cultures without Hg treatment. To ensure the validity of this study the number of fronds in control groups should have a 7-fold increase by the end of 7 days experiment as specified in the ISO 20079 criterion (ISO 20079, 2005). After exponential growth for 7 days, the FN of *L. gibba, L. minor, S. polyrhiza* increased more than 7 times (R^2^ = 0.9966, 0.993, and 0.9857, respectively) (Figures 1A–C) indicating the validity of this study. No significant difference was observed among these three duckweed lines from days 0 to 7 (*P* > 0.05). The relative growth rate (RGR) based on FN was defined as the average specific growth during a certain period, and this was used to assess the growth of three duckweed lines. The RGR of *L. gibba, L. minor* and *S. polyrhiza* in control conditions after 7 days were 0.28 ± 0.003, 0.29 ± 0.007, and 0.28 ± 0.006 per day, respectively (Figures 1D–F). No significant difference was observed among three duckweed lines at days 1, 3, 5, 7 (*P* > 0.05).

### Effect of Hg on the growth of duckweeds

The FN and FW of duckweeds grown in media supplemented with 0.25, 0.5, 1, 2, 4, and 8 mg/L Hg were used to evaluate the toxic effect of Hg on the growth of duckweeds. The FN of all three duckweed lines showed a close relationship with Hg concentration, and in all Hg treatments FN increased with time (Figures 1A–C). The three treated lines showed significant differences in FN when compared with untreated plants at all Hg concentrations analyzed (0.25, 0.5, 1, 2, 4, and 8 mg/L) (*P* < 0.05). No significant difference in FN was observed between *L. gibba* and *L. minor* from days 3 to 7 (*P* > 0.05). However at day 7, the FN of *S. polyrhiza* showed significant differences with *L. gibba* and *L. minor* at 8 mg/L Hg (*P* < 0.05), indicating that *S. polyrhiza* was more resistant to Hg. No significant difference was observed between the 4 mg/L and 8 mg/L treatments (*P* > 0.05) on FN for the three lines, suggesting that all three lines were equally affected at 4 and 8 mg/L levels of Hg.

The RGR values of the three duckweed lines also changed with increasing Hg level and exposure time (Figures 1D–F). In the *L. gibba* line, there was no significant difference between RGR values at 8 mg/L and 4 mg/L treatment from days 1 to 7 (*P* > 0.05) but significant differences were observed between 8 mg/L and 0, 0.25, 0.5, 1 and 2 mg/L treatments at days 3, 5, and 7 (*P* < 0.05; Figure 1D). In *L. minor*, no significant difference was observed between the RGR values at 8 mg/L and 0, 0.25, 0.5, 1, 2, and 4 mg/L treatments at day 1 (*P* < 0.05; Figure 1E). The RGR value at 8 mg/L began to show significant differences with 0, 0.25, 0.5, 1, and 2 mg/L Hg treatments (*P* < 0.05). In *S. polyrhiza*, the RGR value at 8 mg/L treatment was not significantly different from 1, 2, and 4 mg/L till day 5 (*P* > 0.05; Figure 1F) indicating that the negative influence on RGR rate of *S. polyrhiza* was lower than for *L. minor* and *L. gibba*.

In order to allow for the accurate quantification of the inhibitory effect of Hg, the percent inhibition of growth rates (Ir) of three lines was estimated based on FN. This was used to determine the EC50 (half maximal effective concentration) values as well as a dose-response relationship as has been described in the ISO 20079 guidelines (ISO 20079, 2005). When the three duckweed lines were grown at 4 mg/L Hg, the growth inhibition reached 50% at day 3 in *L. gibba*, day 5 in *L. minor* and day 7 in *S. polyrhiza*. The growth inhibition exceeded 50% within 24 h of exposure to 8 mg/L Hg for all three duckweed lines. The highest EC50 values of *L. gibba, L. minor* and *S. polyrhiza* at day 1 were 4.4, 5.0, and 7.5 mg/L, respectively (Table 1). Among the three duckweed lines, the highest EC50 value was obtained in *S. polyrhiza* and the lowest EC50 value was obtained in *L. gibba*. These results indicate that the *S. polyrhiza* line had the highest tolerance to Hg when compared with the *L. minor* and *L. gibba* lines.

**Table 1:** Toxicity assessment of dose-response regression equations for *L. gibba, L. minor* and *S. polyrhiza* under different Hg treatments.

**Species**	**Exposure days**	**Regression equation**	**EC50 (mg/L)**
*L. gibba*	1	y = 1.258lgx-0.806, R^2^ = 0.914	4.4 (2.6, 11.7)
	3	y = 1.226lgx-0.625, R^2^ = 0.92	3.2 (2.0, 6.8)
	5	y = 1.044lgx-0.38, R^2^ = 0.897	2.3 (1.3, 5.0)
	7	y = 1.166lgx-0.291, R^2^ = 0.936	1.8 (1.1, 2.9)
*L. minor*	1	y = 0.926lgx-0.641, R^2^ = 0.989	4.9 (3.6, 7.7)
	3	y = 0.877lgx-0.442, R^2^ = 0.96	3.2 (2.4, 4.7)
	5	y = 0.854lgx-0.384, R^2^ = 0.94	2.8 (2.1, 4.1)
	7	y = 0.919lgx-0.266, R^2^ = 0.912	1.9 (1.1, 3.8)
*S. polyrhiza*	1	y = 1.057lgx-0.928, R^2^ = 0.984	7.5 (5.4, 12.3)
	3	y = 0.88lgx-0.766, R^2^ = 0.966	7.4 (5.0, 13.4)
	5	y = 0.837lgx-0.608, R^2^ = 0.989	5.3 (3.7, 9.0)
	7	y = 0.895lgx-0.454, R^2^ = 0.968	3.2 (2.4, 4.7)

**Figure 2:**
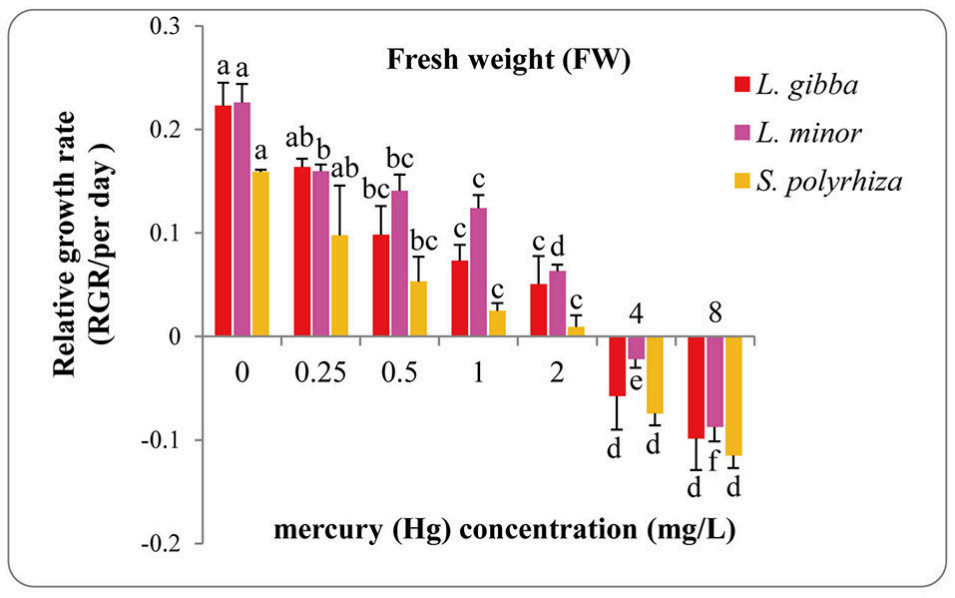
Effects of different concentrations of Hg on the relative growth rates based on fresh weight (FW) of three duckweed lines. The letters (a, b, c, d) on the column graphs indicated Tukey tests analyses results among different Hg treatments in the same duckweed. The same letters indicated no significant differences and different letters indicated significant difference among treatments. Error bars indicated standard deviation.

The FW of treated duckweeds was measured at the end of experiment (day 7) to evaluate the RGR values of different Hg treatments (Figure 2). The RGR based FW of all three lines declined sharply with Hg treatment and even negative growth rates were observed at 4 and 8 mg/L treatments. All of the RGR values based upon FW at 4 and 8 mg/L treatments were found to be significantly different from those at 0, 0.25, 0.5, 1, and 2 mg/L treatments. Significant differences between 4 and 8 mg/L treatment were also observed in *L. minor* (*P* < 0.05) while no significant difference was observed in *L. gibba* and *S. polyrhiza* (*P* < 0.05).

### Effect of Hg on antioxidant protective mechanism

**Figure 3:**
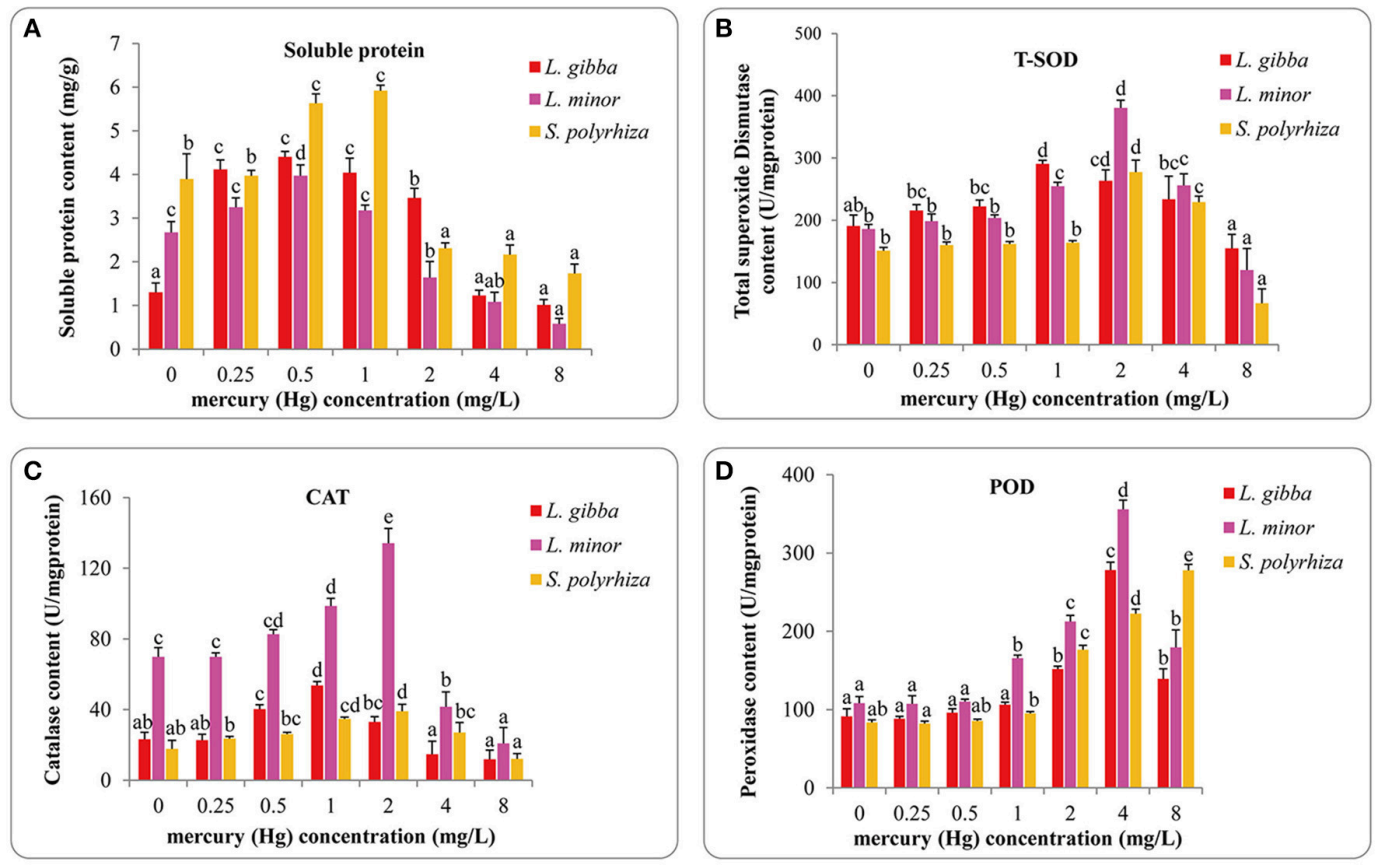
Effects of different concentrations of Hg on the content of soluble protein **(A)** and activities of T-SOD **(B)**, CAT **(C)**, and POD **(D)** of three duckweed lines. Indices were measured after 7 days treatment and calculated based on the fresh weight (FW). The letters (a, b, c, d, e) on the column graphs indicated Tukey tests analyses results among different Hg treatments in the same duckweed. The same letters indicated no significant differences and different letters indicated significant difference among treatments. Error bars indicated standard deviation.

The soluble protein content of the three lines increased with lower levels of Hg (to around 1 mg/L), but was decreased with higher concentrations of Hg (Figure 3A). The highest soluble protein contents reported were 4.40 ± 0.13 mg/g in *L. gibba*, 3.97 ± 0.25 mg/g in *L. minor* at 0.5 mg/L treatment and 5.92 ± 0.13 mg/g in *S. polyrhiz*a at 1 mg/L treatment. Significant differences were observed between the control and 0.25, 0.5, 1, and 2 mg/L treatments in *L. gibba* (*P* < 0.05). No significant difference was observed between the control and either 0.25 or 1 mg/L treatments in *L. minor* (*P* > 0.05) but 0, 0.25, and 1 mg/L treatments were significantly different from 0.5, 2, 4, and 8 mg/L treatments (*P* < 0.05). In the *S. polyrhiza* line, no significant difference was observed between the control and 0.25 mg/L treatment (*P* > 0.05), but the 0 and 0.25 mg/L treatments had significantly different values for soluble protein content from the 0.5, 1, 2, 4, and 8 mg/L treatments (*P* < 0.05).

The total superoxide dismutase (T-SOD) activities increased with Hg treatment in all three lines and reached a peak at 1 mg/L in *L. gibba*, 2 mg/L in *L. minor* and *S. polyrhiza*. The *L. gibba* line had 290.50 ± 5.59 U/mg protein, the *L. minor* line 380.59 ± 12.21 U/mg protein and the *S. polyrhiza* line 277.40 ± 19.16 U/mg protein (Figure 3B). No significant difference was observed between the control and 0.25, 0.5 mg/L treatments in the three lines (*P* > 0.05). However, the 0.25, 0.5 mg/L treatments were significantly different from 1 and 2 mg/L treatments (*P* < 0.05) in *L. gibba*. In *L. minor*, the 0, 0.25, and 0.5 mg/L treatments were significantly different from 1, 2, 4, and 8 mg/L treatments (*P* < 0.05). And in *S. polyrhiza*, only the 0, 0.25, and 0.5 mg/L treatments were significantly different from 2, 4, and 8 mg/L treatments (*P* < 0.05).

As well as investigating T-SOD activity, we also investigated variation in the levels of catalase (CAT) activity (Figure 3C). The highest levels observed were 53.64 ± 2.23 U/mg protein in *L. gibba* at 1 mg/L Hg treatment, 134.11 ± 8.42 U/mg protein in *L. minor* and 39.11 ± 3.91 U/mg protein in *S. polyrhiza* at 2 mg/L Hg treatment. The lowest activities recorded at 8 mg/L treatment were 11.92 ± 5.16 U/mg protein in *L. gibba*, 20.86 ± 9.03 U/mg protein in *L. minor* and 12.17 ± 3.01 U/mg protein in *S. polyrhiza*. In *L. gibba*, there was no significant difference between the control and 0.25, 2, 4, and 8 mg/L treatments (*P* > 0.05). In *L. minor*, significant differences were observed between the control and 1, 2, 4, and 8 mg/L treatments (*P* < 0.05). And in *S. polyrhiza*, significant differences were only observed between control and 1, 2 mg/L treatments (*P* < 0.05).

We finally investigated variation in the levels of peroxidase activity (POD) (Figure 3D). We saw maximum POD level at 4 mg/L treatment in *L. gibba* and *L. minor*, and like the previous protective mechanisms the activity was reduced at higher concentrations. In contrast POD activity increased consistently in the *S. polyrhiza* line until the highest concentration tested (8 mg/L). This indicates that *S. polyrhiza* exhibits a constant response to high levels of Hg. The highest value obtained in *L. gibba, L. minor* and *S. polyrhiza* were 278.36 ± 9.93, 355.77 ± 11.85, and 277.94 ± 7.41 U/mg protein, respectively. No significant difference was observed within the control, 0.25 and 0.5 mg/L treatments of the three lines (*P* > 0.05). However, the 0, 0.25, and 0.5 mg/L treatments were significantly different from 2, 4, and 8 mg/L treatments in all three lines (*P* < 0.05). All of the POD activity measurements in 1 mg/L were significantly different from 2 and 4 mg/L treatments. However, there was no significant difference between 0.5 and 1 mg/L treatments in *L. gibba* and *S. polyrhiza* (*P* > 0.05). In *L. minor*, significant differences were observed between 1 mg/L and 0, 0.25, and 0.5 mg/L treatments (*P* < 0.05).

### Chlorophyll content

**Figure 4:**
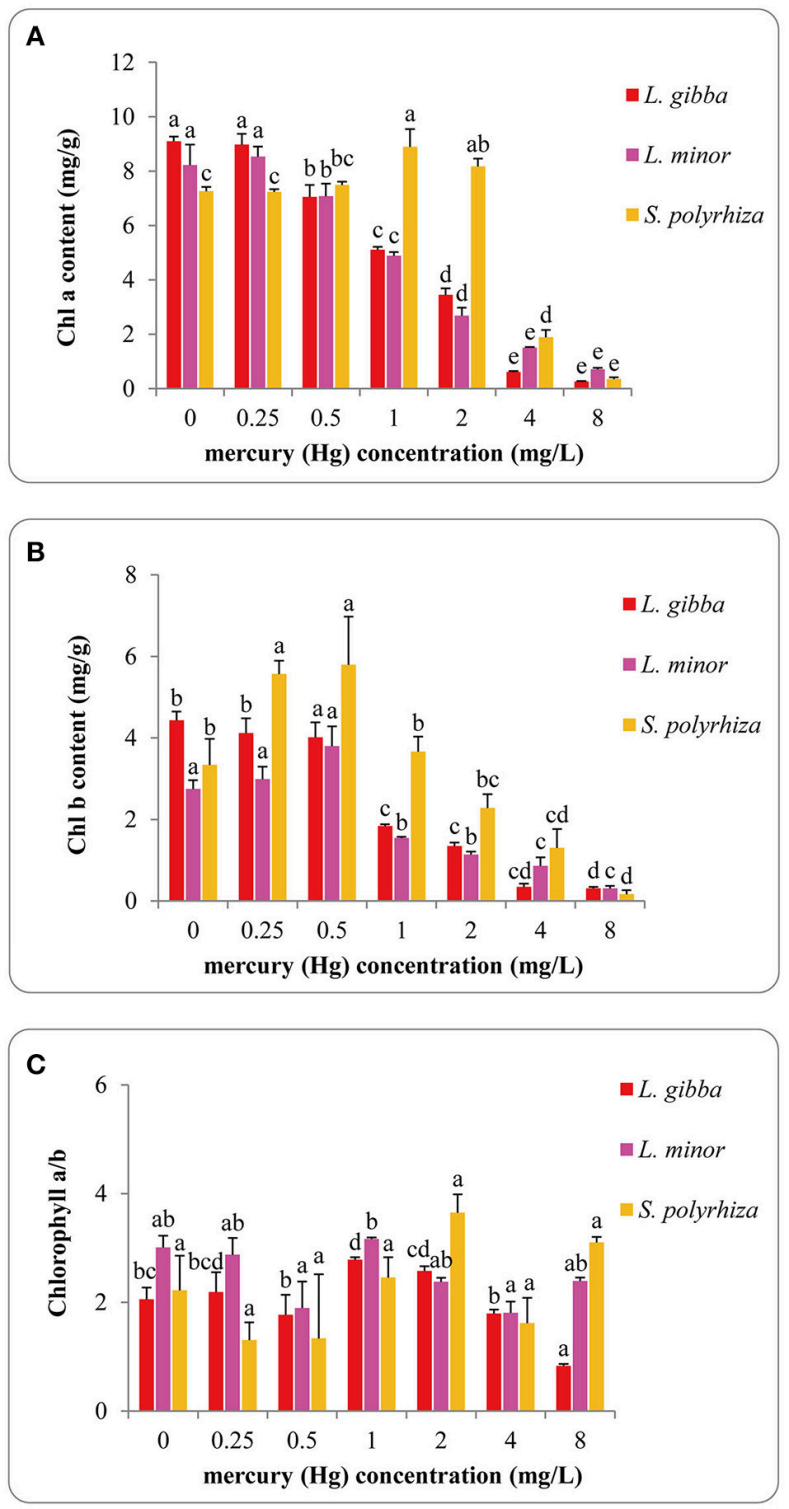
Effects of different concentrations of Hg on the content of Chlorophyll a **(A)**, Chlorophyll b **(B)** and Chlorophyll a/b **(C)** of three duckweed lines. The pigment content was measured after 7 days Hg treatment and calculated based on the fresh weight (FW). The letters (a, b, c, d, e) on the column graphs indicated Tukey tests analyses results among different Hg treatments in the same duckweed. The same letters indicated no significant differences and different letters indicated significant difference among treatments. Error bars indicated standard deviation.

The influence of Hg on photosynthetic pigment content (chlorophyll *a*, chlorophyll *b* and chlorophyll *a*/*b*) of *L. gibba, L. minor*, and *S. polyrhiza* lines is shown in Figures 4A–C. The pigment content in *L. gibba* was negatively correlated with Hg exposures and the highest values of chlorophyll *a*, chlorophyll *b* and chlorophyll *a*/*b* were 9.09 ± 0.18 mg/g (FW), 4.43 ± 0.22 mg/g (FW), and 2.78 ± 0.11 mg/g (FW) in control groups. In *L. minor*, the Chlorophyll *a* content reached to its maximum at 0.25 mg/L Hg treatment of 8.53 ± 0.37 mg/g (FW), while the maximum content of chlorophyll *b* was 3.80 ± 0.49 mg/g (FW) at 0.5 mg/L treatment. In *S. polyrhiza*, the maximum chlorophyll *a* content was 8.89 ± 0.65 mg/g (FW) at 1 mg/L treatment, whilst chlorophyll *b* content reached to the maximum value of 5.79 ± 1.18 mg/g (FW) in the 0.5 mg/L treatment. The highest ratios of chlorophyll *a*/*b* were 2.78 ± 0.11, 3.17 ± 0.14 for *L. gibba* and *L. minor* at 1 mg/L Hg treatment and 3.65 ± 0.69 for *S. polyrhiza* in the 2 mg/L treatment.

The chlorophyll *a*, chlorophyll *b* and chlorophyll *a*/*b* content of *L. gibba* were significantly different between the control and 1, 2, 4, and 8 mg/L treatments (*P* < 0.05). No significant difference was observed between the control and 0.25 mg/L Hg treatment (*P* > 0.05). In *L. minor*, chlorophyll *a* and chlorophyll *b* content in control samples were significantly different from 0.5, 1, 2, 4, and 8 mg/L treatments (*P* < 0.05). In *S. polyrhiza*, chlorophyll *a* content in the control was significantly different from 1, 2, 4, and 8 mg/L treatments (*P* < 0.05). There were significant differences between the chlorophyll *b* content of the control and 0.5, 4, 8 mg/L treatments (*P* < 0.05). No significant difference was observed among all the treatments of chlorophyll *a*/*b* (*P* > 0.05).

### Effects of Hg on starch content

**Figure 5:**
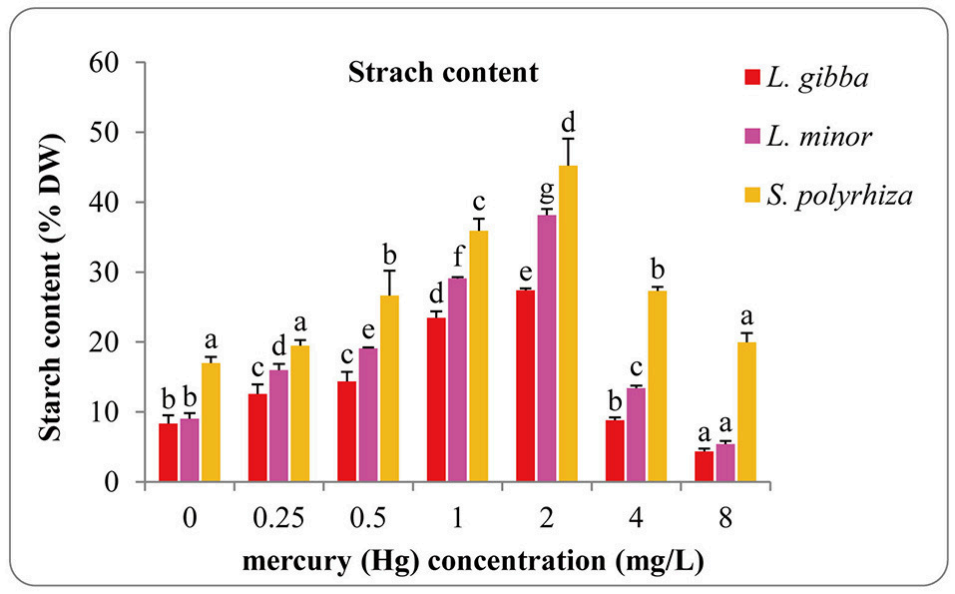
Effects of different concentrations of Hg on starch content of three duckweed lines. The starch content was measured after 7 days and calculated based on the dry weight (DW). The letters (a, b, c, d, e, f) on the column graphs indicated Tukey tests analyses results among different Hg treatments in the same duckweed. The same letters indicated no significant differences and different letters indicated significant difference among treatments. Error bars indicated standard deviation.

Starch content increased with increasing Hg concentration in all three lines and reached the highest in the 2 mg/L treatment with the maximal values of 27.42 ± 0.24, 38.16 ± 0.86, and 45.24 ± 3.86 (% DW) for *L. gibba, L. minor*, and *S. polyrhiza*, respectively (Figure 5). In every treatment the starch content of *S. polyrhiza* was higher than that of *L. gibba* and *L. minor*. Significant differences were observed between control and 0.25, 0.5, 1, 2, and 8 mg/L treatments in *L. gibba* (*P* < 0.05). In *L. minor*, significant differences were observed among all Hg treatments (*P* < 0.05). No significant difference was observed between the control and 0.25 mg/L treatment in *S. polyrhiza* (*P* > 0.05), whilst the control was significantly different from 0.5, 1, 2, 4 mg/L treatments (*P* < 0.05).

### Mercury accumulation in duckweed

**Figure 6:**
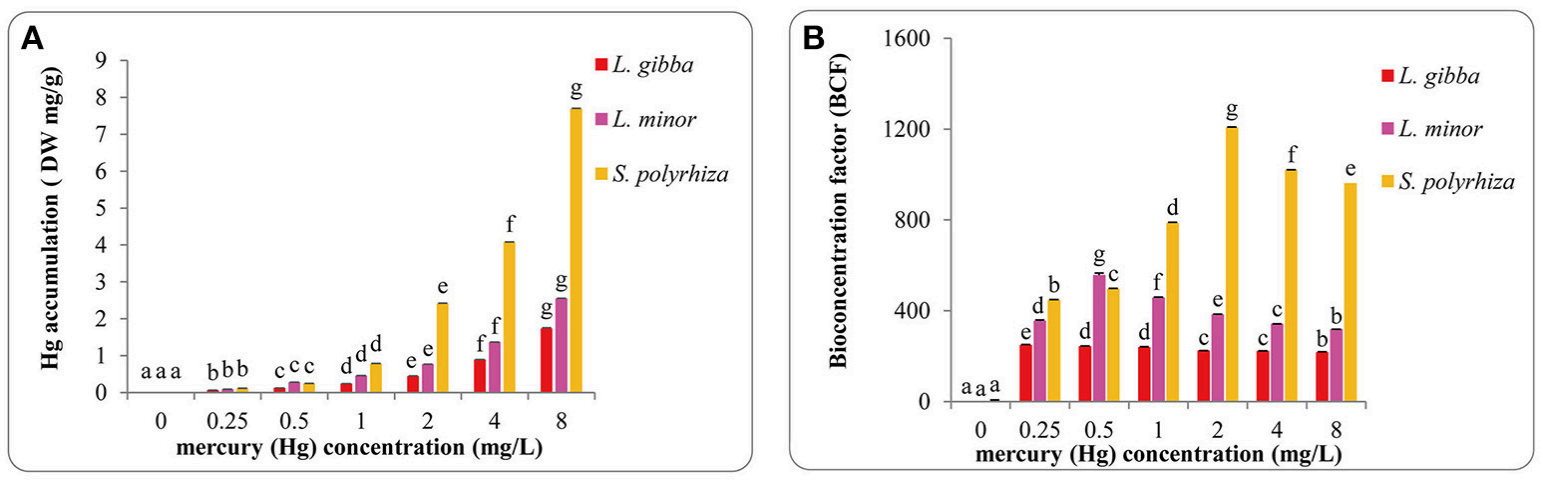
Hg accumulation **(A)** and bioconcentration factors (BCF) of Hg accumulation **(B)** of three duckweed lines under different concentrations of Hg treatments. Hg accumulation was measured after 7 days and calculated based on the dry weight (DW).The letters (a, b, c, d, e, f, g) on the column graphs indicated Tukey tests analyses results among different Hg treatments in the same duckweed. The same letters indicated no significant differences and different letters indicated significant difference among treatments. Error bars indicated standard deviation.

Mercury accumulation was monitored in all three lines at the various concentrations (Figures 6A,B). In all lines, as expected Hg accumulation increased sharply with Hg treatments and reached the maximum in the 8 mg/L treatment (Figure 6A). The highest values in *L. gibba, L. minor*, and *S. polyrhiza* were 1.74 ± 0.02 mg/g (DW), 2.55 ± 0.004 mg/g (DW), and 7.70 ± 0.01 mg/g (DW) respectively, showing that the *S. polyrhiza* line can accumulate substantially more Hg. Hg accumulation was significantly different among every treatments in the three lines (*P* < 0.05). *S. polyrhiza* clearly accumulated the highest Hg quantity followed by *L. minor* and *L. gibba*.

It is important to relate the concentration of certain elements within an organism to the concentration in the environment where the organism exists, and this can be done by measuring the bioconcentration factor (BCF). We calculated BCF values of Hg accumulation in all treatments (Figure 6B). In *L. gibba*, BCF dropped from the maximum of 249.42 ± 2 in the control to 217.47 ± 2 in the 8 mg/L treatment. In *L. minor*, the value increased to the highest at 557.52 ± 8 in the 0.5 mg/L treatment and then decreased in treatments between 1 and 8 mg/L. In *S. polyrhiza*, the highest value obtained was 1208.67 ± 2.41 in the 2 mg/L treatment. No significant difference was observed between treatments of 0.5 and 1 mg/L as well as 2 and 4 mg/L in *L. gibba* (*P* > 0.05). However in *L. gibba*, the BCF values at 0.5, 1, 2, and 4 mg/L treatments showed significant differences from 0.25 and 8 mg/L treatments (*P* < 0.05). BCF values in *L. minor* and *S. polyrhiza* were significantly different among every treatment (*P* < 0.05).

## Discussion

### *L. gibba, L. minor*, and *S. polyrhiza* are ideal plants for Hg biomonitoring and bioremediation

Previous studies have reported that duckweeds are highly sensitive to a broad range of pollutants and show multivariable stress responses when compared with other aquatic macrophytes (Cedergreen et al., 2004). Their characteristics of simple structure, minute size, rapid multiplication and easy cultivation make them ideally suited for use as bioindicators in aquatic habitats (Wang, 1990; Forni, 2014). In such assays, the use of visible parameters, such as total frond number make it possible to assess contamination in a direct and rapid manner. Easily measurable stress response parameters such as starch content, or photosynthetic pigment can also be used to provide effective toxicological evaluations (USEPA, 1996; Marwood et al., 2001; Baumann et al., 2008; Pietrini et al., 2015) and individual contaminants may elicit a more specific response in such assays. In this study, we investigated three lines corresponding to different duckweed species using a combination of growth and chemical assays. We report serious growth effects at high Hg levels (4 and 8 mg/L), and consistent with other current research, we suggest that duckweeds provide a suitable system for biomonitoring of Hg in waters contaminated with less than 4 mg/L Hg level.

Our results indicated that out of the three lines analyzed, *L. gibba* was more suitable for Hg biomonitoring than *L. minor* and *S. polyrhiza* as it displayed the highest sensitivity to Hg. It should be noted that these results are specific for Hg, and different sensitivities of duckweeds have been reported for other heavy metals. For example, Lahive et al. (2011) reported that *Landoltia punctata, L. minor, Wolffia brasiliensis* and *L. gibba* had distinct sensitivity to zinc sulfate with *L. punctata* being the most sensitive. Gür et al. (2016) reported that *L. minor* was more sensitive to boron (B) than *L. gibba* while *L. gibba* showed a wider range of responses for B than *L. minor* in biomonitoring. Lakatos et al. (1993) also reported that *L. minor* was more sensitive than *L. gibba* to both copper and Bonion biocide exposure. According to Leblebici and Aksoy (2011) the ability of *L. minor* to extract lead from the surrounding environment was more effective than *S. polyrhiza* while *S. polyrhiza* was more sensitive than *L. minor*. According to Appenroth et al. (2010) *S. polyrhiza* was more sensitive to nickel than *L. minor*. These findings demonstrate distinctly that the outcome of biomonitoring assessments of pollutants using duckweeds is both highly species dependent, but also dependent on the contaminant. Therefore, selection of duckweed species for toxicity assessment should be done carefully.

Our results indicated BCF was a more effective measurement than Hg accumulation to provide accurate quantification of heavy metal uptake in duckweeds. It has been previously been shown for a variety of plants that higher BCF values indicate a stronger ability for metal uptake (Salt et al., 1995; Zayed et al., 1998). Accordingly, in our experiments, the *S. polyrhiza* line had a relatively high BCF value. According to Zayed et al. (1998), a plant with a BCF of over 1,000 can be considered as good accumulator for the compound of interest. Therefore, the results obtained here suggest that the *S. polyrhiza* line used in this study could potentially be used for Hg remediation in aquatic ecosystems. Whilst both *L. gibba* and *L. minor* are unsuitable for Hg bioremediation, these lines used in this study would be more effective for use in Hg biomonitoring.

### Antioxidant substances, photosynthetic pigment and starch content are ideal biomarkers in toxicity assessments

A range of protective substances, including antioxidant enzymes (SOD, CAT, POD), soluble proteins, flavonoids, and other phenolics have been shown to accumulate in plants as an adaptive mechanism for coping with certain stresses (Horling et al., 2003; Mittler et al., 2004; Sharma and Dietz, 2009; Varga et al., 2013). Each enzyme has a different role to play in plant protection; SOD converts superoxide to H_2_O_2_ (Mishra et al., 2006), whilst CAT and POD breakdown the H_2_O_2_ (Scandalios et al., 1994). Some soluble proteins also form an important antioxidant constituent that is needed to maintain metabolism (Singh and Tewari, 2003). Flavonoids and other phenolics may constitute a secondary antioxidant system when antioxidant enzymes are depleted, and specifically counter the oxidant load in the vacuole (Agati et al., 2012).

In this manuscript we report that the three duckweeds tested resist metal-stress at low Hg treatments (0.25, 0.5,1 mg/L) by accumulating soluble protein within their tolerance range. In all cases the soluble protein content decreased at high Hg levels (2, 4, 8 mg/L). These findings are similar to the changes in soluble protein content in *L. minor* reported under treatments with between 0 and 500 μM CdCl_2_ (Razinger et al., 2008). However, Varga et al. (2013) have reported direct reduction of protein content in *L. minor* and *L. gibba* after 24 h exposure to different Hg and Cd concentrations (0, 100, 200, 300, 400, 500, and 600 μM), most likely due to excessive stress in the short-term. The inability to synthesize protein at high Hg concentrations (2, 4, 8 mg/L) might be due to several reasons, such as shortage of energy, carbohydrates or a reduced level of nutrients essential for protein synthesis, such as Mg and K (Mazhoudi et al., 1997; Gardea-Torresdey et al., 2004; Wang et al., 2008), increased protease activity (Palma et al., 2002) or structural changes induced by DNA damages or damage to the photosynthetic system (Ates et al., 2004; Gardea-Torresdey et al., 2004). The changes that we observed in the levels of SOD, CAT and POD in Hg-treated duckweeds were similar to findings in other studies, following the oxidative stress by Hg treatment in wheat (Sahu et al., 2012) and into ammonium-induced oxidative stress in *L. minor* (Huang et al., 2013). Our studies revealed that the *S. polyrhiza* line had a higher tolerance to Hg than both the *L. gibba* and *L. minor* lines, since antioxidant enzyme activities in this line decreased at relatively high Hg levels and POD content increased until 8 mg/L.

It has been reported that heavy metal stress affects pigment content in many plant species (Prasad et al., 2001; Hou et al., 2007; Perreault et al., 2013; Sree et al., 2015). In this study, the pigment content of *L. minor* and *S. polyrhiza* showed an initial increase, followed by a significant decrease under different Hg treatments, and this is likely due to the combinational effect of photoprotection and antioxidative production as reported by Lalau et al. (2015). *L. gibba* was more sensitive to Hg than both *L. minor* and *S. polyrhiza*, and accordingly we observed that the pigment content decreased under Hg stress from 0.25 to 8 mg/L. Moreover, the chlorophyll *a* content decreased sharply at high Hg concentrations (from 2 to 8 mg/L) indicating that chlorophyll *a* was a more sensitive biomarker than chlorophyll *b* and chlorophyll *a*/*b*, a phenomenon that has been observed in other studies (Hou et al., 2007; Appenroth et al., 2010). Starch accumulation has also been identified as a protective mechanism in duckweeds to overcome adverse environmental conditions (Sree and Appenroth, 2014). Accordingly, we observed an increase in the starch content of all three duckweed lines when exposed to low Hg treatments (0.25, 0.5, 1, 2 mg/L). In a similar manner to other research, we observed that the inhibition of growth also reduced the demand for carbohydrates and consequently reduced the starch content (Appenroth et al., 2010). Moreover, we observed substantial reduction in pigment content when duckweeds were exposed to high Hg treatments (4, 8 mg/L) and this is likely to lead to insufficient supplies of carbohydrates. As a result the starch reserves are likely to have been used to fulfill the carbohydrate demand (Sree et al., 2015).

Collectively our data show that different concentrations of mercuric chloride (HgCl_2_) affected the growth, photosynthetic pigment, starch content and antioxidant system of *L. gibba, L. minor* and *S. polyrhiza*. When Hg concentrations are increased, we observed reduction of RGR, FN, FW in all three lines. As Hg concentrations are altered, the levels of starch, photosynthetic pigment and soluble protein are all modulated. Moreover, at these concentrations, increasing the synthesis of SOD, CAT, and POD appears to enhance the antioxidant protective mechanisms. However, at very high levels of Hg stress, these mechanisms have been inhibited. Our analysis of EC50 values indicated that the *L. gibba* line was more sensitive to Hg toxicity than both *L. minor* and *S. polyrhiza*. Therefore it can be concluded that *L. gibba* is more efficient in Hg biomonitoring than *L. minor* and *S. polyrhiza. S. polyrhiza* shows a very high BCF (over 1,000) and based on this we propose that the *S. polyrhiza* line used in this study has a great potential for bioremediation of Hg contaminated aquatic ecosystems.

## Ethics statement

This manuscript did not include human subjects or animals. Therefore, ethics approval was not required.

## Author contributions

HH designed and funded this project. JY and GL conducted the experiments. PH and SH cultivated plants and processed sample treatments. YC and ZW performed statistical analysis and data processing. AB, SK, PD, and LY wrote the manuscript.

### Conflict of interest statement

The authors declare that the research was conducted in the absence of any commercial or financial relationships that could be construed as a potential conflict of interest. The handling Editor and reviewer, KA, declared their involvement as co-editors in the Research Topic, and confirm the absence of any other collaboration.
